# The Gender and Geographic Location of Authors Impact Citation Rates of *GBE* Papers

**DOI:** 10.1093/gbe/evag090

**Published:** 2026-04-27

**Authors:** David Alvarez-Ponce

**Affiliations:** Biology Department, University of Nevada, Reno, NV, USA

## Introduction

Even though their representation in academia has increased in the last decades, women are still underrepresented, especially at top STEM departments and senior positions (Ceci et al. 2009; Ceci et al. 2014; Holman et al. 2018; Baker and Koedel 2024; Alvarez-Ponce and Vesper 2025a, 2025b). Potential reasons include women receiving less support to conduct their research, and their achievements being less recognized and visible (Damschen et al. 2005; Goulden et al. 2011; Martin 2012; Shor et al. 2015; Ross et al. 2022; Samaniego et al. 2023). In most fields, women accumulate fewer citations throughout their careers than their male peers (Long 1992; Davenport and Snyder 1995; Duch et al. 2012; Huang et al. 2020; Wu 2024). An important question is whether, on average, female-authored research articles are more or less cited than male-authored ones. The answer seems to depend on the specific discipline: whereas female-authored articles are cited at similar rates or even more cited in many fields (Long 1992; Symonds et al. 2006; Duch et al. 2012; Pautasso 2013; Card et al. 2020; Huang et al. 2020; Wu 2024), the opposite has been found in other fields (Aksnes et al. 2011; Ferber and Brün 2011; Larivière et al. 2013; Maliniak et al. 2013; Chatterjee and Werner 2021; Meho 2022; Teich et al. 2022; Alvarez-Ponce and Vesper 2025b). This underscores the importance of evaluating the gender citation gap separately within each discipline.

There are geographical biases as well. Research produced in the Global South tends to be less cited than research produced in the Global North (Meneghini et al. 2008; Di Bitetti and Ferreras 2017; Gomez et al. 2022; Liu et al. 2023; Nakamura et al. 2023; Atwell et al. 2026; Herman et al. 2026; Sağnıç et al. 2026). Potential reasons include researchers based in the Global South having access to fewer resources, and their research being sometimes perceived as less important or of inferior quality. However, the effect of geographic location on citation rates is again discipline-specific, underscoring the importance of evaluating it separately within each field.

Women and/or authors based in the Global South being to some extent excluded from the scientific discourse is not only unfair but also detrimental to science and society as a whole: their perspectives, approaches, and findings are important for the advancement of science and for the betterment of society. *Genome Biology and Evolution* (*GBE*) is deeply committed to promoting gender and geographic equity and in the last years has been monitoring to what extent gender affects acceptance rates, time to first decision, and citation rates (Eyre-Walker and Katz 2022, 2024). Continuing with these monitoring efforts, here we analyze all articles published in *GBE* from its launch in 2009 until the end of 2025 to evaluate the effect of authors’ gender and geographic location on articles’ citation rates. We show that articles with female first authors and those with corresponding authors based in the Global South tend to receive fewer citations. We discuss potential reasons and mitigating strategies.

## Methods

For each article published in *GBE* as of December 2025 (n = 3762), we obtained the following information: article title, article type, name of the first author, name of the corresponding author, country/territory of affiliation of the corresponding author, date of publication, and number of citations. We removed 194 articles of special types that are often not cited at the same rates as regular articles (editorials, editor's notes, highlights, meeting reports, biographies, corrections/corrigenda, and errata), resulting in 3568 articles.

We then augmented the dataset by adding the following information. First, we searched the first names of all first authors and all corresponding authors in the Genderize.io database (last accessed: August 18, 2024) in order to classify them as “probably woman” (if >90% of the individuals with that name in the database were women), as “probably man” (if >90% of the individuals with that name in the database were men), “unisex name” (if individuals in the database did not identify as either women or men >90% of the time), or “unknown” (if the name was not present in the database). For simplicity, throughout this manuscript, we use the terms “female author” and “male author” to denote “probably female author” and “probably male author”, respectively. We also use the terms “female author” and “male author” as synonyms of “woman author” and “man author”, respectively. We acknowledge that our method is not appropriate to classify nonbinary, gender fluid, gender neutral, and gender nonconforming individuals, and that it can misgender some individuals (however, the inferences produced by our method have been shown to be accurate in most cases; e.g. Alvarez-Ponce and Vesper 2025a; Holman et al. 2018). Second, for each corresponding author's country/territory, we obtained the most recent GDP per capita from the World Bank (https://data.worldbank.org/indicator/NY.GDP.PCAP.CD; last accessed on April 26, 2025) and classified them as part of the Global North or the Global South according to the UN Trade and Development (UNCTAD)'s classification. Finally, for each article, we computed the number of citations per year as the number of total citations divided by 2026 minus the publication year.

All statistical analyses were conducted in R v.4.4.3. Prior to ANCOVA analysis, we transformed the number of citations per year by adding 0.1 to all values and log-transforming them to improve normality.

## Results

### Women's Representation Among *GBE* Authors

**Fig. 1.**
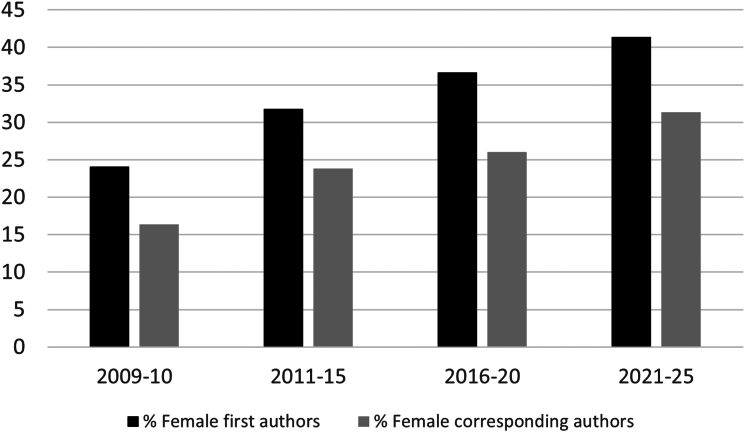
Representation of women among *GBE* authors.

Out of the 3568 articles in the dataset, 1,075 had female first authors, 1,877 had male first authors, 519 had first authors with unisex names, and 97 had first authors of unknown gender (Fig. 1). In addition, 823 had female corresponding authors, 2,250 had male corresponding authors, 395 had corresponding authors with unisex names, and 100 had corresponding authors with names of unknown gender. For each quinquennium since the journal started being published in 2009, we calculated the fraction of female authors as the number of female authors divided by the number of authors whose gender could be inferred (women + men), separately for first and corresponding authors. The results show that women's representation has steadily increased since 2009, but that women have always been, and continue to be, underrepresented among both kinds of authors, and particularly among corresponding authors (Fig. 1). These results are in agreement with previous results from different fields of Biomedicine and the Life Sciences, including Evolutionary Biology (Débarre et al. 2018; Holman et al. 2018; Edwards et al. 2018; Fox et al. 2019; Rushworth et al. 2021; Alvarez-Ponce and Vesper 2025a, 2025b; Alvarez-Ponce et al. 2026).

### Articles With Male First Authors Receive More Citations

**Fig. 2.**
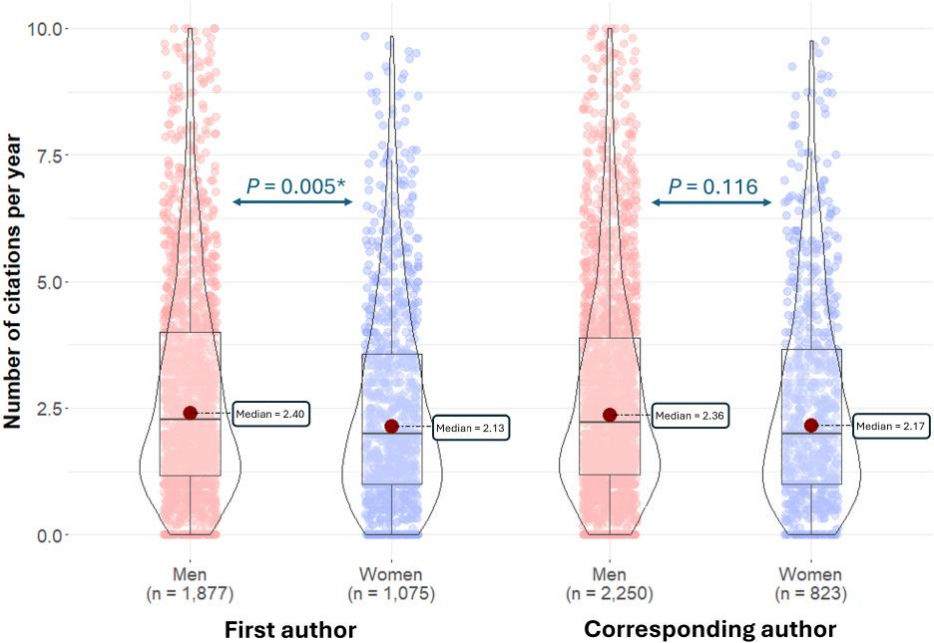
Number of citations per year for articles with female versus. male first and corresponding authors. Values above 10 were not included in the figure. *, statistically significant differences (*P* < 0.05).

On average, *GBE* articles with female first authors were cited 3.04 times per year (median: 2.13), whereas those with male first authors were cited 3.32 times per year (median: 2.40; Fig. 2). A Mann–Whitney *U* test found statistically significant differences (*P* = 0.005). Conversely, we found no significant differences between articles with female corresponding authors (average: 3.06, median: 2.17) and articles with male corresponding authors (average: 3.27, median: 2.36; *P* = 0.116; Fig. 2).

Among the top-709 most cited articles (those with 4.69 or more citations per year), 196 had female first authors, 397 had male first authors, 159 had female corresponding authors, and 452 had male corresponding authors. Among the bottom-634 least cited articles (those with less than one citation per year), 218 had female first authors, 303 had male first authors, 156 had female corresponding authors, and 376 had male corresponding authors. The Top-709 and the bottom-634 articles significantly differed in the fraction of first authors that are women (33.1% vs. 41.8%; Fisher's exact test, *P* = 0.003); however, the 2 sets did not differ in the fraction of corresponding authors that are women (26.0% vs. 29.3%; *P* = 0.232).

### Articles Whose Corresponding Authors are based in the Global North Receive More Citations

**Fig. 3.**
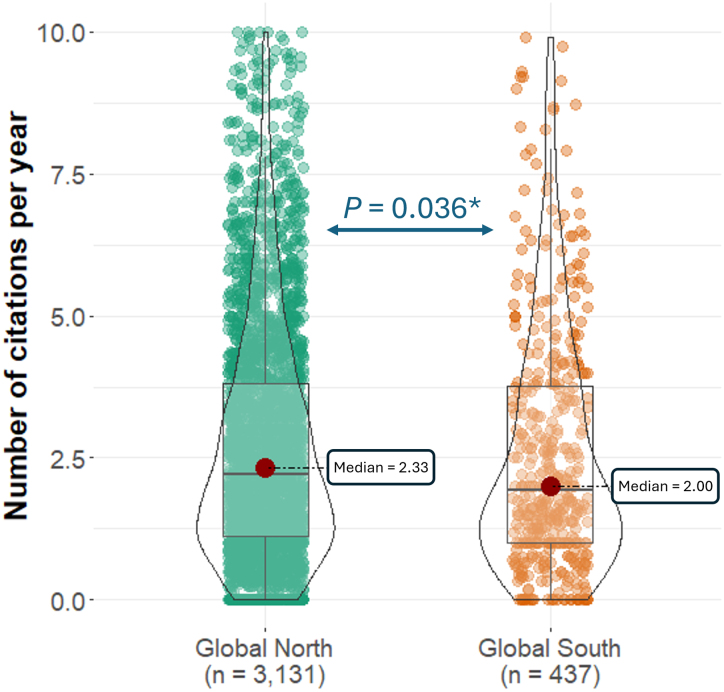
Number of citations per year for articles with corresponding authors based in the global north versus. the Global South. Values above 10 were not included in the figure. *, statistically significant differences (*P* < 0.05).

A Kruskal–Wallis test showed that the country/territory in which the corresponding author is based has a significant effect on the number of citations per year (*P* = 0.008). Articles whose corresponding authors are based in the Global North tend to be more cited (average: 3.23, median: 2.33 citations/year, *n* = 3131) than those whose corresponding authors are based in the Global South (average: 2.94, median: 2.00 citations/year, *n* = 437; Mann–Whitney *U* test, *P* = 0.036; Fig. 3). However, no significant correlation was found between article's number of citations per year and the GDP per capita of the country/territory of the corresponding author (Spearman's rank correlation coefficient, ρ = 0.023, *P* = 0.176).

### Multivariate Analysis Confirms our Observations

We conducted an ANCOVA using the number of citations per year as the dependent variable, and the gender of the first author, the gender of the corresponding author, and the location of the corresponding author (Global North vs. Global South) as independent variables. Both the gender of the first author and the location of the corresponding author had significant effects on the number of citations per year (*P* = 0.008 and *P* = 0.989, respectively), whereas the gender of the corresponding author had no significant effect (*P* = 0.993).

## Discussion

A number of explanations have been proposed for why male-authored articles tend to be more cited than comparable female-authored articles in some fields. First, male researchers tend to amass a higher number of publications throughout their careers since they publish more articles per year and/or they tend to have longer academic careers (Duch et al. 2012; Huang et al. 2020); authors would thus be more familiar with their names and would be more likely to cite them (the so-called “fast-food effect”; Kelly and Jennions 2006). Second, as a result of their higher number of accumulated publications, men tend to have larger coauthor networks, and coauthors tend to cite each other (Mcdowell and Smith 1992; Rothstein and Davey 1995; Bosquet and Combes 2013; Ductor et al. 2023). Third, on average, male researchers are more likely than women to occupy positions of prestige in academia, and the work of highly prestigious academics tends to enjoy more visibility (the so-called “Matthew effect”; Merton 1968). Fourth, men self-cite more often than women (Fowler and Aksnes 2007). Fifth, researchers may preferentially cite authors of the same gender, thus putting women at a further disadvantage in most disciplines (Dion et al. 2018). Sixth, men are more likely than women to publish in highly prestigious journals (Krishnamurthy et al. 2017; Bendels et al. 2018; Shen et al. 2018); however, this cannot be a driver of the gender citation gap reported here, since all analyzed articles were published in the same journal. Last, women's scientific contributions may be systematically undervalued (the so-called “Matilda effect”; Knobloch-Westerwick and Glynn 2013; Knobloch-Westerwick et al. 2013).

Similar explanations can be invoked to explain why articles by authors based in the Global North tend to be more cited: their work tends to be more valued, and they tend to accumulate more publications, to have larger collaboration networks, to occupy more influential positions in academia, to cite each other, and to publish in highly prestigious journals (Cash-Gibson et al. 2018; Collyer 2018; Ekdale et al. 2022; Espinosa-Rada and Ortiz 2022).

Our results somewhat differ from those of a previous analysis of the effect of author's gender on *GBE* articles’ citation rates (Eyre-Walker and Katz 2024). The differences probably stem from the different datasets and analytical approaches used in the 2 studies. First, they analyzed a smaller sample of articles (those published between 2009 and the first issue of 2022). Second, they counted the number of citations 2 years and 5 years after publication, while we counted all citations accumulated since publication. While they found that articles with female first authors were significantly less cited than articles with male first authors in the first 2 years after publication, they found no differences after 5 years.

To increase the citation rates of articles by female first authors and by authors based in the Global South, journals can promote articles from these groups on their websites, commission highlights, and push for press releases. In addition, academic societies can promote such authors by preferentially accepting their applications to give oral presentations and/or by inviting them to give talks. Nonetheless, ultimately, improvements will also require more profound, structural changes in academia as a whole.

## Data Availability

All data used in this study are publicly available.
